# The benefit of vaccination against COVID-19 outweighs the potential risk of myocarditis and pericarditis

**DOI:** 10.1007/s12471-022-01677-9

**Published:** 2022-03-09

**Authors:** T. A. Klamer, M. Linschoten, F. W. Asselbergs

**Affiliations:** 1 Department of Cardiology, Division of Heart and Lungs, University Medical Centre Utrecht, Utrecht University, Utrecht, The Netherlands; 2 Institute of Cardiovascular Science, Faculty of Population Health Sciences, University College London, London, UK; 3 Health Data Research UK and Institute of Health Informatics, University College London, London, UK

**Keywords:** COVID-19, COVID-19 vaccination, Coronavirus disease 2019, Myocarditis, Pericarditis, Side effect

## Abstract

Vaccines against coronavirus 2019 disease (COVID-19) have shown to be greatly effective in preventing viral spread, serious illness and death from this infectious disease and are therefore critical for the management of the COVID-19 pandemic. However, the listing of myocarditis and pericarditis as possible rare side effects of the messenger RNA (mRNA) vaccines against COVID-19 by regulatory agencies has sparked discussion on the vaccines’ safety. The most important published cohort studies to date demonstrat that myocarditis is a very rare side effect after COVID-19 mRNA vaccination, with an incidence of approximately 1–4 cases per 100,000 vaccinated persons. Young males (16–29 years) appear to be at highest risk, predominantly after receiving the second dose. The disease course is self-limiting in a vast majority of cases: 95% of patients show a rapid resolution of symptoms and normalisation of cardiac biomarkers, electro- and echocardiographic findings within days. Importantly, the available data suggest that the incidence rate of myocarditis in the context of COVID-19 is much greater than the risk of this side effect following vaccination. We conclude that the benefit of vaccination against COVID-19 outweighs the potential risk of myocarditis and pericarditis in both adolescents and adults. Prospective follow-up of patients who have developed these complications after vaccination is required to assess long-term outcomes.

## Introduction

In the summer of 2021, six months after the Emergency Use Authorization of the messenger RNA (mRNA) vaccines against coronavirus 2019 disease (COVID-19) from Pfizer-BioNTech and Moderna, the European Medicines Agency (EMA) and the American Food and Drug Administration (FDA) recommended listing myocarditis and pericarditis as new side effects [1, 2]. Prior to this recommendation, a number of cases of myocarditis and pericarditis had been reported in the scientific literature, as well as in the Vaccine Adverse Event Reporting System and EudraVigilance databases [1–4]. After reviewing the available data, both the EMA and FDA concluded that the benefit of vaccination outweighs the risk of myocarditis and pericarditis and therefore soon advised to continue the vaccination programme in the beginning of July 2021 [1–3]. However, both regulatory authorities also recommended vigilance.

New data on the occurrence of these side effects has recently appeared [5–10], reigniting the discussion on the safety of COVID-19 vaccines, especially on social media. In this point of view paper, we summarise the findings of the most important peer-reviewed population studies published thus far on the occurrence of myocarditis and pericarditis after COVID-19 vaccination, to assess whether the benefit of vaccination outweighs the possible risk of these adverse events.

## Myocarditis, pericarditis and COVID-19

Myocarditis is a rare disease with an estimated annual incidence of 16 per 100,000 persons in the general population [11]. The true incidence may be higher, as signs and symptoms vary, and it therefore can be challenging to make the diagnosis [12, 13]. Viruses are the primary cause of myocarditis, including amongst others adeno- and enteroviruses. Recently, severe acute respiratory syndrome coronavirus 2 (SARS-CoV-2) has been associated with myocarditis as well, and multiple cases have been described since the outbreak of the COVID-19 pandemic [14, 15].

The CAPACITY-COVID collaborative consortium recently published data on more than 16,000 patients hospitalised with COVID-19 across 18 countries, of whom 0.2% and 0.15% were diagnosed with myocarditis or pericarditis during admission in a routine care setting, respectively [16]. Similar findings were reported by the Centers for Disease Control and Prevention (CDC) in the United States, where 0.15% of the patients with COVID-19 were diagnosed with myocarditis during an inpatient or outpatient hospital encounter across more than 900 hospitals [17]. In patients with COVID-19, the risk ratio for myocarditis was 15.7 (95% confidence interval (CI) 14.1–17.2) after adjustment for hospital and patient characteristics compared with a COVID-19-negative cohort that visited a hospital in the same time period [17].

When competitive athletes were screened for cardiac complications to determine if they could safely return to play after COVID-19 using screening protocols that included a cardiac magnetic resonance (CMR) imaging, 2.3% of the athletes were diagnosed with clinical or subclinical myocarditis after a recent infection [18]. However, only 5 out of 1,597 athletes (0.3%) had both symptoms associated with myocarditis and abnormal laboratory (troponin level) and/or imaging findings (electrocardiography (ECG) and/or echocardiography) [18]. In a systematic review of CMR imaging studies performed in athletes with a history of COVID-19, the proportion of athletes with a confirmed diagnosis of myocarditis varied between 0% and 15% [19]. In the general population, data on the occurrence of subclinical myocarditis and pericarditis after COVID-19 are lacking.

Histopathological studies on cardiac tissue of patients with SARS-CoV‑2 and myocarditis have infrequently demonstrated findings corresponding with typical viral myocarditis such as diffuse lymphocyte infiltration in the heart [4, 20]. It is therefore hypothesised that excessive activation of the immune system contributes to the development of myocarditis or pericarditis in certain patients with a genetic predisposition [4, 21].

## Myocarditis, pericarditis and COVID-19 vaccines

Vaccines are the most critical tool in efforts to control the COVID-19 pandemic. The dramatic impact of the pandemic accelerated the development and approval of two mRNA vaccines: Comirnaty from Pfizer-BioNTech and Spikevax from Moderna [22–24]. After the start of the vaccination campaign in Israel, its Ministry of Health started active surveillance of myocarditis in February 2021 following early reports on the occurrence of myocarditis as a possible adverse side effect [6]. The first case series in the literature appeared three months later and described three young male patients who had been diagnosed with myocarditis two days after the second dose of the Pfizer-BioNTech vaccine [25].

The pathophysiological mechanisms behind the development of myocarditis and pericarditis after a COVID-19 vaccination are currently not completely understood. One hypothesis is that the immune system detects the mRNA molecules as antigens, triggering an immune reaction in certain individuals [4, 26]. Another mechanism that has been proposed is that antibodies against a part of the SARS-CoV-2’s spike protein that the mRNA encodes for, cross-react with structural similar host proteins in the heart, also known as molecular mimicry [27]. Similar mechanisms have been described for the occurrence of neurological phenomena after vaccination against COVID-19, including transverse myelitis [28].

The first large population cohort studies that were conducted in Israel provided more insight into the overall incidence of myocarditis after COVID-19 vaccination. Mevorach et al. used the database of the Israeli Ministry of Health to assess the occurrence of myocarditis from December 2020 until May 2021 [6]. Over 9 million Israeli residents were included, of whom more than 5.4 million had received at least one dose of the Pfizer-BioNTech vaccine. Possible cases were identified based on the *International Classification of Diseases, Nineth Revision* (ICD-9) codes for myocarditis and reviewed case by case to determine the likelihood of the diagnosis. In total, 136 patients were diagnosed with definite or probable myocarditis, mainly within proximity of the second vaccination, corresponding with an overall cumulative incidence of 3.83 per 100,000 in males and 0.46 per 100,000 in females. The highest incidence was found in males aged 16–19 years, equalling an incidence of 15.07 per 100,000 young males (Tab. 1) [6]. The age- and sex-adjusted standardised incidence ratio for myocarditis between those that had received a second dose compared with a pre-pandemic cohort was 5.34 (95% CI 4.48–6.40). This difference was mainly driven by young male vaccine recipients (16–19 years of age), in whom the standardised incidence ratio was 13.60 (95% CI 9.30–19.20). The standardised incidence ratio after the first dose was 1.42 (95% CI 0.92–2.10) for the whole population and 1.62 (95% CI 0.32–4.72) for young males (16–19 years). Of the identified 136 cases, 129 (94.9%) had a mild disease course defined as a resolution of symptoms within days with normalisation of cardiac biomarkers and any abnormalities detected with ECG and echocardiography. In one case (0.7%), myocarditis was fulminant and led to death [6].

**Table 1:** Incidence (rate) of myocarditis after COVID-19 vaccination based on data from published large population cohort studies

	Dose	Mevorach et al. [6]^a^					Witberg et al. [7]^b^			Husby et al. [5]^c^						Oster et al. [9]^d^										Chua et al. [8]^e^	
Age category		16–19 years	20–24 years	25–29 years	30–39 years	All ages	16–29 years	≥ 30 years	All ages	12–17 years		12–39 years		All ages		12–15 years		16–17 years		18–24 years		25–29 years		30–39 years		12–17 years	All ages
Vaccine type		P	P	P	P	P	P	P	P	M	P	M	P	M	P	M	P	M	P	M	P	M	P	M	P	P	P
*Both sexes*	Both doses	NA	NA	NA	NA	NA	5.5 (3.6–7.4)	1.1 (0.7–1.6)	2.13 (1.6–2.7)	NA	1.0 (0.2–3.0)	5.7 (3.3–9.3)	1.6 (1.0–2.6)	4.2 (2.6–6.4)	1.4 (1.0–1.8)	NA	NA	NA	NA	NA	NA	NA	NA	NA	NA	18.5 (11.7–29.0)	NA
Male	First dose	1.3	1.9	1.2	0.4	0.6	10.7 (6.9–14.5)	2.1 (1.2–3.0)	4.1 (3.0–5.3)	NA	NA	NA	NA	6.3 (3.6–10.2)	1.5 (1.0–2.2)	NA	0.7 (0.5–1.0)	NA	0.7 (0.5–1.2)	1.1 (0.8–1.5)	0.4 (0.2–0.6)	0.5 (0.3–0.9)	0.2 (0.1–0.4)	0.3 (0.2–0.5)	0.1 (0.0–0.1)	5.6 (2.4–12.5)	NA
	Second dose	15.1	10.9	7.0	3.7	3.8										NA	7.1 (6.2–8.1)	NA	10.6 (9.2–12.2)	5.6 (4.7–6.7)	5.2 (4.6–6.0)	2.4 (1.8–3.3)	1.7 (1.3–2.3)	0.8 (0.6–1.1)	0.7 (0.5–1.0)	37.3 (27.0–51.3)	NA
*Female*	First dose	0.0	0.0	0.0	0.0	0.1	0.3 (0.0–1.0)	0.2 (0.0–0.5)	0.2 (0–0.5)	NA	NA	NA	NA	2.0 (0.7–4.8)	1.3 (0.8–1.9)	NA	0.0 (0.0–0.2)	NA	0.1 (0.0–0.3)	0.1 (0.0–0.3)	0.0 (0.0–0.1)	0.0 (0.0–0.3)	0.0 (0.0–0.2)	0.1 (0.0–0.2)	0.1 (0.0–0.2)	1.1 (0.2–6.6)	NA
	Second dose	1.0	2.2	0.0	0.2	0.5										NA	0.6 (0.4–1.0)	NA	1.1 (0.7–1.7)	0.7 (0.4–1.1)	0.4 (0.3–0.7)	0.8 (0.5–1.3)	0.2 (0.1–0.5)	0.1 (0.0–0.2)	0.1 (0.0–0.2)	4.8 (1.9–11.4)	NA

*P* Pfizer-BioNTech vaccine Comirnaty, *M* Moderna vaccine Spikevax, *NA* not available

^a^ Incidence per 100,000 vaccinated persons (95% confidence interval (CI) not available) within 21 days after first and second vaccine dose; interval between first and second dose: 21 days

^b^ Incidence per 100,000 vaccinated persons (95% CI) within 42 days after first vaccine dose; interval between first and second dose: 21 days

^c^ Incidence per 100,000 vaccinated persons (95% CI) within 28 days after first or second vaccine dose; median interval between first and second dose: 35 days

^d^ Incidence per 100,000 doses administered (95% CI) within 7 days after first and second vaccine dose; interval between first and second dose: not available. Since original results were calculated per 1,000,000 doses administered, the incidence was divided by ten to calculate the incidence per 100,000 doses administered

^e^ Incidence per 100,000 vaccinated persons (95% CI) within 14 days after first and second vaccine dose; interval between first and second dose: not available

The second Israelian study used comparable methods and searched for cases in the database of Clalit Health Services, the largest health care organisation of Israel providing care to 4.7 million Israeli residents [7]. The occurrence of myocarditis in this cohort was similar to that in the study of Mevorach et al. [6], with an overall cumulative incidence of 2.13 per 100,000 persons [7]. Again, myocarditis was most often observed in young males aged 16–29 years (10.69 cases per 100,000 vaccinated persons) (Tab. 1). Overall, almost all cases (53/54, 98.1%) were classified as mild or moderate [7, 13]. Left ventricular dysfunction was detected in 29% of the patients diagnosed with myocarditis. Two-thirds of patients were discharged from hospital without ongoing medical treatment, and cardiac function had normalised in all patients who underwent additional testing after discharge. Only one patient developed fulminant myocarditis leading to cardiogenic shock.

Husby et al. evaluated data from the entire Danish population, comprising almost 5 million individuals, by linking the Danish Vaccination Register to hospital-based diagnoses from the Danish National Patient Register [5]. The primary outcome measure of this study was defined as a myocarditis and/or pericarditis diagnosis (according to the ICD-10 codes) with the co-occurrence of troponin release and a length of hospital stay of at least one day. The association between COVID-19 vaccination and cardiac arrest or death was a secondary outcome measure. Among vaccinated individuals in Denmark (84.3% of the population), 83.8% were vaccinated with Comirnaty (Pfizer-BioNTech) and 12.0% with Spikevax (Moderna). The incidence rate of myocarditis was 1.7 per 100,000 vaccinated persons (*n* = 269 cases). Those vaccinated with Spikevax had a statistically significantly higher risk of myocarditis during follow-up than unvaccinated individuals (hazard ratio (HR) 3.92, 95% CI 2.30–6.68), especially among individuals aged 12–39 years (HR 5.24, 95% CI 2.47–11.12). Similar findings were reported by Patone et al. [10]. In line with the Israelian studies, myocarditis was diagnosed more frequently in men (73%) in the Danish population [5]. Of the myocarditis patients, 40% were aged 12–39 years, and the clinical outcomes after myocarditis were generally described as mild without any readmissions or the occurrence of heart failure or death within 28 days after vaccination. Importantly, individuals vaccinated with either mRNA vaccine had a markedly reduced risk of cardiac arrest or death compared with unvaccinated individuals.

**Fig. 1:**
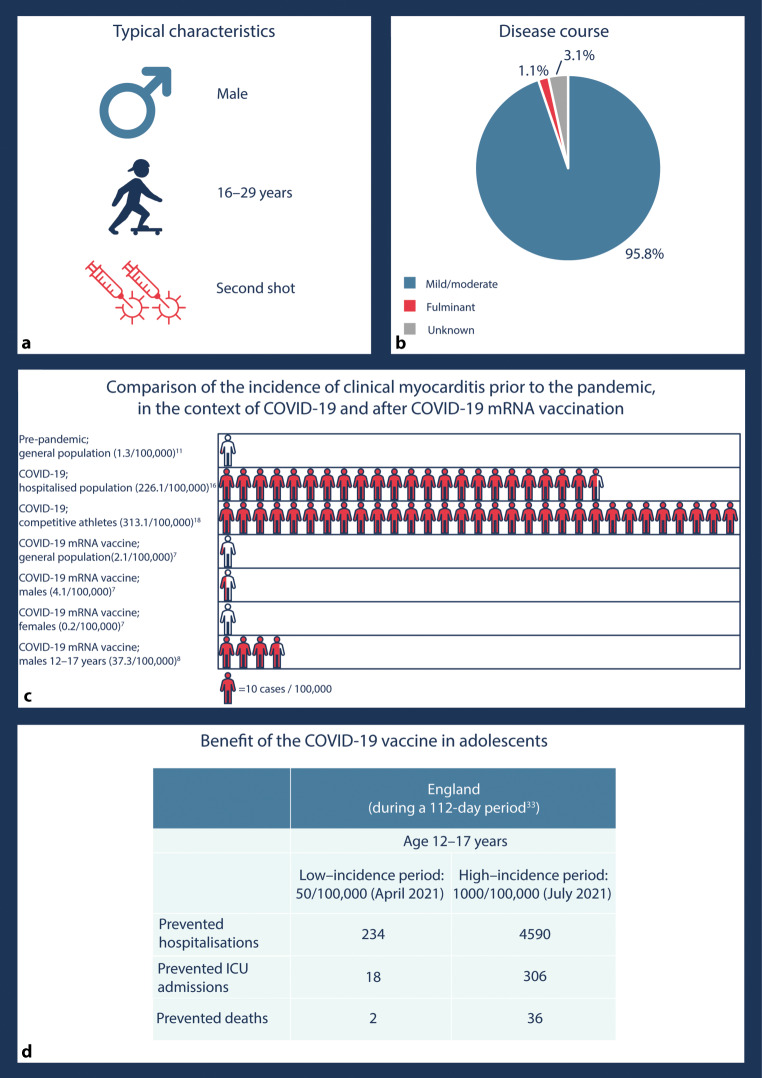
COVID-19-vaccine related myocarditis. **a** Typical characteristics of individuals developing myocarditis after COVID-19 mRNA vaccination. Studies showed a marked age and sex difference: incidence of myocarditis is 5.0–17.5 times higher in young males aged 16–29 years [5–10]. Myocarditis mainly occurs after second vaccine dose [6–8]. **b** Number of patients with a mild/moderate, fulminant or unknown disease course of myocarditis; frequencies were calculated based on two studies that reported these data [6, 7]. **c** Clinical myocarditis after COVID-19 vaccination was most often diagnosed within 14–30 days after the first or second dose. For comparative purposes, annual incidence of myocarditis prior to the COVID-19 pandemic was assumed to be evenly spread each year, with overall annual incidence of 16 cases per 100,000 persons [11], which equals 1.3 cases per 100,000 persons per month. Based on joint data from CAPACITY-COVID registry and Lean European Open Survey on SARS-CoV‑2 (LEOSS), 37 of 16,368 SARS-CoV‑2 infected patients were diagnosed with myocarditis during median length of hospital stay of 9 days (interquartile range 5–18) [16]. Of 1597 competitive athletes screened for safe return to play after COVID-19, 5 were diagnosed with clinical myocarditis (cardiac symptoms present at the time of cardiac testing) 13–77 days after the infection [18]. **d** Estimated benefit of COVID-19 mRNA vaccination in young males (12–17 years) in England in April 2021 (low incidence of SARS-CoV‑2 infection) and July 2021 (high incidence of SARS-CoV‑2 infection)

The findings of five large cohort studies [5–9] and four publicly-accessible databases collecting data on adverse events after vaccination [29–31] are summarised in Tab. 1 and 2, respectively. Typical characteristics of patients diagnosed with myocarditis after mRNA vaccination and a comparison of the incidence of clinical myocarditis prior to the pandemic in patients with COVID-19 and after COVID-19 mRNA vaccination are visualised in Fig. 1.

**Table 2:** Incidence of myocarditis after COVID-19 vaccination based on data from voluntary vaccine adverse event reporting systems

	The Netherlands^a^				United Kingdom^b^	Europe^c^	United States^d^
Sex	Male		Female		Male and female	Male and female	Male and female
Dose	First dose	Second dose	First dose	Second dose	First and second doses	At least one dose	First and second doses
*Pfizer-BioNTech (Comirnaty)*	3.0	3.8	0.6	1.1	4.3	4.2	6.5
*Moderna (Spikevax)*	10.0	10.9	5.9	6.4	14.7	6.2	3.7

^a^ Incidence per 1 million vaccine doses; results based on data from the Netherlands Pharmacovigilance Centre Lareb (until 19 October 2021) [29]

^b^ Incidence per 1 million vaccine doses; results derived from website of the United Kingdom government (until 28 July 2021, accessed on 20 December 2021) [30]

^c^ Incidence per 1 million vaccinated persons; results based on data from EudraVigilance (until 6 August 2021), calculated by Lane et al. [31]

^d^ Incidence per 1 million vaccinated persons; results based on Vaccine Adverse Event Reporting System data (until 6 August 2021), calculated by Lane et al. [31]

## Risks versus benefits

Being aware of the risks of myocarditis and pericarditis following COVID-19 mRNA vaccination, do the benefits of vaccination outweigh these risks, especially in young males? According to an analysis conducted by the CDC, administering a million second doses of an mRNA vaccine in males aged 12–17 prevents 5700 COVID-19 cases, 215 hospitalisations, 71 admissions to the intensive care unit and 2 deaths over a period of 17 weeks [32]. Not surprisingly, the benefit of the vaccines in older patients is even larger. Similar estimations were computed in England (Fig. 1d) [33].

Furthermore, in a study by Barda et al., the risk ratio of myocarditis in the context of a SARS-CoV‑2 infection was estimated to be 18.28 (95% CI 3.95–25.12) compared with the uninfected group, which corresponds with a risk difference of 10.96 events per 100,000 patients (95% CI 5.57–15.80) [34]. This illustrates that the incidence of myocarditis in the context of COVID-19 is much greater than the risk of this side effect following COVID-19 vaccination.

## Conclusion

The benefits of the COVID-19 mRNA vaccines clearly outweigh the slim risks of myocarditis and pericarditis, with an overall cumulative incidence of 0.6–4 and 0–2 per 100,000 vaccinated persons for males and females, respectively. Prospective studies evaluating long-term outcomes in patients who have developed these complications, as well as the safety of administering a subsequent COVID-19 mRNA vaccine dose, are still warranted.
